# Assessment of Association of Increased Heart Rates to Cardiovascular Events among Healthy Subjects in the United States: Analysis of a Primary Care Electronic Medical Records Database

**DOI:** 10.5402/2011/924343

**Published:** 2011-04-28

**Authors:** Carl V. Asche, Jaewhan Kim, Amit S. Kulkarni, Paula Chakravarti, Karl-Erik Andersson

**Affiliations:** ^1^Department of Pharmacotherapy, University of Utah College of Pharmacy, Salt Lake City, UT 84112, USA; ^2^Department of Physical Therapy, University of Utah College of Health, Salt Lake City, UT 84112, USA; ^3^Center on Aging, University of Utah, Salt Lake City, UT 84112, USA; ^4^Division of Public Health, University of Utah School of Medicine, Salt Lake City, UT 84108, USA; ^5^Novartis Pharmaceuticals Corporation, East Hanover, NJ 07936, USA; ^6^Wake Forest University, Winston-Salem, NC 27104, USA

## Abstract

*Objective*. To determine whether increases in heart rates (HRs) over time leads to adverse cardiovascular (CV) events among “healthy subjects.” *Methods*. This retrospective cohort study used the GE Centricity EMR database. “Healthy subjects” were defined as those with Charlson Comorbidity Index (CCI) score = 0 and Chronic Disease Score (CDS) = 0 at baseline. Subjects were followed for 3 years post the first date of a clinical encounter between the patient and provider. Those aged ≥18 years old with baseline HR and ≥2 post-index HR readings were identified between 01/01/1996 to 03/30/2007. *Results*. There were 93,952 “healthy subjects” at baseline (median age 42 years; 67.2% women; mean HR was 75.8 (SD: 11) bpm); 20.7% with a mean HR at baseline of 76.3 (SD: 11.3) bpm (median age 45; 63 women) experienced a CV event during 3 years of follow-up. The mean HR was higher among those with a CV event (76.3 bmp) compared to those without a CV event (75.7 bpm). A Cox regression model indicated that an increase in HR by 5 bpm was associated with a 1% increase in CV event risk. *Conclusions*. Elevated HRs are associated with an increased likelihood of CV events among “healthy subjects”.

## 1. Introduction

Several studies on different patient population shave linked high heart rates (HRs) and cardiovascular (CV) mortality and morbidity [1–4]. Different approaches have been used; however, there seem to be no studies based on data from individuals without comorbidities obtained from “real-world” primary care settings and using national electronic medical records from large databases. 

Measuring comorbidity brings about its' own set of unique challenges as it sometimes requires the researcher to detect a difference between different treatment groups. De Groot et al. [5] listed a number of reasons as to why one needs to measure comorbidity: (1) to strengthen internal validity by correcting for confounding, (2) to predict study outcomes, (3) to identify effect modification, and (4) to improve upon statistical efficiency by consolidating multiple occurring comorbidities into one variable [5]. There are a number of comorbidity scores available, the most common of which is to summarize the comorbidity information into an index or score. 

The purpose of this study was to evaluate whether a large electronic medical record (EMR) database, based on primary care data, could be used to determine whether increases in HRs over 3-year followup leads to adverse CV clinical outcomes among “healthy subjects” identified by the Charlson Comorbidity Index (CCI) and Chronic Disease Score (CDS). 

## 2. Methods

### 2.1. Data Source

This retrospective cohort study used the General Electric (GE) Centricity (GE Healthcare, GE Healthcare IT, Princeton, NJ) a deidentified Health Insurance Portability and Accountability Act of 1996 (HIPPA)—compliant EMR database. At the time of the study (2007), the EMR contained data on more than 8.9 million patients. The GE EMR database is comprised of data submitted by more than 70 consortium member institutions located in more than 40 states. Consortium members represent a variety of practice types, ranging from solo practitioners to community clinics, academic medical centers, and large integrated delivery networks. Approximately, two thirds of the participating clinicians identify themselves as primary care physicians. The dataset is comprised of submitted longitudinal patient data and includes but is not limited to demographic information, payment type (commercial, Medicare, Medicaid, self-pay, and unrecognized payment type), enrollment dates, vital signs, laboratory orders and results, medication list entries and prescriptions, *International Classification of Diseases, *9th* Revision, (ICD-9) Clinical Modification* (*ICD-99-CM*) diagnosis codes, and Current Procedure Terminology Version 4 (CPT-4), indicating the type of procedure if provided.

### 2.2. Study Design and Inclusion Criteria

We conducted a retrospective cohort study using the GE EMR database. The study period ranged from 01/01/1996 to 03/30/2007. “Healthy subjects” were defined as subjects with a lower risk (i.e., zero) of mortality at baseline based on validated and widely used comorbidity indices (CCI and CDS). CCI is based upon ICD-9-CM codes. On the other hand, CDS is based on prescription information. The “healthy subjects” were identified by having a CCI and CDS of 0 at baseline. The index date was defined as the first Health Maintenance Organization (HMO) activity. CCI and CDS scores were calculated based on data available in the 395 days prior to the index date. These healthy subjects were followed for 3 years post-index date. “Healthy subjects” aged 18 years of age and older with a baseline HR (closest to before index date or on index date) and at least two post-index HR readings were identified between 01/01/1996 to 03/30/2007. For “healthy subjects” who experienced a CV event, all HR readings prior to the CV event were considered (i.e., HR readings on the day of the CV event were excluded from analysis). CV events were identified by the ICD-9-CM codes (250.xx, 393.xx–398.xx, 401.xx–405.xx, 410.xx, 414.xx–417.xx, 420.xx, 429.xx–438.xx, 440.xx–448.xx, 451.xx–458.xx, 582.xx-583.xx, 585.xx-586.xx, 588.xx-589.xx, 746.xx-747.xx, 785.1–785.3). The “healthy subjects” identified were continuously followed in the EMR during the study observation period (395 days prior to index date to 3 years post-index date). All HR readings and CCI and CDS (measured at first, second, and third year post-index period) were included in this model as time varying covariates. 

As mentioned, the overall comorbidity burden was assessed using the CCI and CDS. The CCI is the most commonly used index to measure and summarize health conditions. The CCI contains 19 categories of comorbidity, defined primarily using ICD-9 diagnosis codes [6, 7]. Each category has an associated weight based on the adjusted risk of 1-year mortality. The overall comorbidity score reflects the cumulative increased likelihood of 1-year mortality. CCI has been popularly used and validated in research [8–10]. 

Another commonly used comorbidity index is the CDS. The CDS uses pharmacy dispensation information for specific drug classes to estimate the burden of comorbidities [11]. CDS is a risk-adjusted metric based on age, gender, and history of dispensed drugs. This score is an aggregate comorbidity measure based on medication use. It has been proven to be valid in predicting hospitalization, health resource utilization, and mortality [12–17]. The CDS is the sum of all chronic diseases identified from drug therapies over the full follow-up period.

### 2.3. Statistical Analysis

After defining and selecting subjects who met the inclusion and exclusion criteria, descriptive statistics were utilized to describe the baseline population and unadjusted outcomes overall. Using a Kaplan-Meier curve, the survival probability adjusted by heart rate of subjects overtime was shown (Figure 2). In order to consider the association between CV events and changes in HR over 36 months along with other covariates after index date, a Cox proportional hazard regression with an outcome that is time to CV event anytime during 3-year followup was used. Among covariates, HR, age, CCI, and CDS were included as time-varying covariates. All statistical analysis was performed at a significance level of.05 using STATA v.10 (StataCorp, College Station, TX. This project was reviewed and approved by the University of Utah institutional review board.

## 3. Results

### 3.1. Patient Cohorts

**Figure 1:**
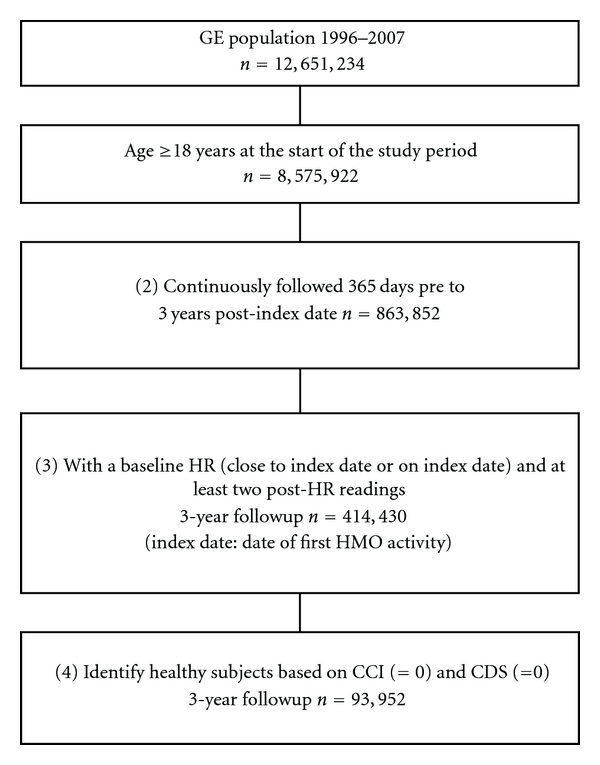
Selection of subjects. Notes: GE: General Electric; EMR: Electronic Medical Record; HR: Heart Rate; CCI: Charlson Comorbidity Index; CDS: Chronic Disease Score; HMO: Health Maintenance Organization.

**Figure 2:**
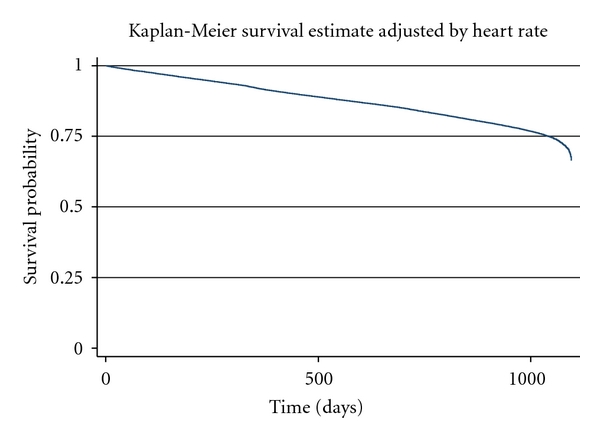
Kaplan-Meier survival estimate adjusted by heart rate over 3 years follow-up (*n* = 93,952).

From the GE EMR database between the years 1996 and 2007, with 3-year followup from index date, 93,952 healthy subjects (Figure 1) were selected (CCI = 0 and CDS = 0). The mean age of subjects at baseline was 42 (SD: 9.7) years old, and approximately 67% were female (Table 1). Among those, 32.4% were Caucasian, approximately 60% had commercial health insurance, and 10.6% were current smokers. Mean HR and BMI of the healthy subjects at baseline were 75.8 bpm (SD: 11.0) and 28.2 (SD: 6.4) kg/m^2^ (Table 1). 

A total of 19,445 healthy subjects (20.7% of 93,952; mean age 44.4 (SD: 9.4); 63% women) experienced a CV event anytime during 3-year followup (Table 2). Their mean follow-up time by CV event was 510 (SD: 313) days after the index date. The mean HR in this group was 76.3 bpm (SD: 11.3). There were 74,507 subjects (79.3% out of 93,952; mean age: 41.4 (SD: 9.7); 68% women) with 3 years of followup who did not experience a CV event (censored). Mean HR in this group was 75.7 (SD 10.9) bpm (Table 2). The group without CV events as compared to the group with CV events were younger (41 years versus 44 years; *P* value <.001) and more were female (68% versus 63%; *P* value <.001).

### 3.2. CV Events during 3-Year Followup

Based on the information on the subjects who had and had not CV events during 3-years followup, factors related to CV events were identified in Table 3. Most importantly, an increase in HR by 1 bpm was associated with a 0.3% increase in relative risk for a CV event. Exponentially, this equates to an increase in HR by 5 bpm to be associated with a 1% increase in relative risk for a CV event. In addition, a one-year increase in age raised the hazard ratio by 2.4%. African-American and Hispanic subjects had a higher risk of having CV events as compared to Caucasian subjects by 38% and 42%, respectively. Increases in post-index CCI and CDS were associated with an increase in hazard ratio by 49% and 22% over 3-year followup, respectively. Non-smokers had a lower risk of having CV events than current smokers by approximately 5%. 

## 4. Discussion

This study examined whether increases in HRs over a 3-year follow-up period leads to adverse CV clinical outcomes among “healthy subjects” identified by CCI and CDS in the United States using the GE EMR database. 

HR is a commonly measured clinical parameter. However, the importance of HR may not be generally considered in treatment decisions. A recent study by Andersson et al. found that patients with overactive bladder (OAB), a substantial portion of whom had an increased HR at baseline, were more likely to experience CV comorbidities than patients without OAB. In addition, these OAB patients were more likely to be exposed to medications with antimuscarinic effects, potentially causing a further increase in HR. This study found that 39.1% of OAB patients started on an antimuscarinic medication had an HR over 80 bpm [18]. These results suggest that HR and CV comorbidities may not be considered when making treatment decisions regarding OAB. 

Evidence does exist that suggests HR may influence patient outcomes and mortality. Animal studies suggest that there is a relatively constant number of total heart beats in a mammal's lifetime, resulting in an inverse relationship between lifespan and HR [19]. Benetos et al. found that after adjusting for major risk factors, men with an elevated HR (>80 bpm) have a decreased probability (>40%) of reaching 85 years of age compared to men with a low HR (<60 bpm) [20]. Epidemiologic studies have found a relationship between elevated HR and mortality. In an analysis of the data from the NHANES Epidemiologic Follow-up Study, elevated HR was found to be associated with increased coronary heart disease and mortality rates [21]. In patients with coronary artery disease (CAD), increased resting HR was found to be an independent predictor for all-cause and cardiovascular mortality [22]. Increased HR in hypertensive patients has also been shown to increase mortality [23].

This study examined whether increases in HR overtime lead to CV clinical outcomes among “healthy subjects” defined by CCI and CDS of zero in the US using an EMR database. This study found that an increase in HR over 3-year followup was significantly related to CV events, showing that HR is an independent predictor of CV events among healthy subjects controlling for potential confounders. Possible mechanisms for this deleterious effect includes accelerated atherosclerosis, increased arterial rigidity, increased myocardial oxygen consumption, decreased myocardial perfusion due to shortened diastole, and increased sympathetic and decreased parasympathetic tone [24, 25].

This study should be considered in light of some limitations. First, this study was based on an EMR; hence, innate limitations of this type of database, such as coding errors and systematic factors upcoding, may impact the results of the study. Second, there might be an underreporting issue because the dataset was from primary care providers. This underreporting might bring about a biased result. Third, we used CCI and CDS only to define healthy subjects. However, the study cohort might have been different had we used different comorbidity measures to identify healthy subjects. Lastly, a weakness associated with a comorbidity index based on a retrospective claims database is that they are usually primarily for reimbursement purposes as opposed to research and, therefore, have varied quality [26, 27].

Large long-term hypertension studies have determined that healthy populations with increased HRs are at increased risk of CV events and overall mortality. Our retrospective cohort study using the GE EMR database also indicates that among “healthy subjects,” elevated HRs are associated with an increased likelihood of CV events. Our study implies that modification of HR is beneficial for patient care, especially in patients with higher risk due to hypertension, diabetes, myocardial infarction, or CV-related chronic illness. Treatment interventions that lower HR such as beta-blockers and certain calcium channel blockers might be beneficial to those with higher HR. In addition, due to the potential risks associated with an increased HR, physicians should be cautious when prescribing medications that have to potential to increase a patient's HR. 

## Figures and Tables

**Table 1:** Baseline characteristics of subjects included (*n* = 93,952).

Variables	Characteristics		
	*N*/Mean	%/SD	Median
Age (as continuous)	42.02	9.68	42
18 to 30	14.98		
31 to 40	29.27		
41 to 50	32.18		
51 to 64	23.57		
≥65	0.00		
Gender			
Female	67.22		
Male	32.78		
Race/Ethnicity			
White	32.38		
Black	4.33		
Hispanic	1.29		
Other	1.95		
Unknown	60.05		
Insurance type			
Commercial	59.58		
Medicare	5.4		
Medicaid	2.04		
Self-pay	1.48		
Unknown	31.49		
Smoking status			
Never	26.49		
Ever	11.61		
Current	10.56		
Unknown	51.34		
Baseline clinical parameters			
Heart rate	75.84	10.98	76
Total cholesterol	198.97	36.57	197
HDL	54.16	15.88	52
LDL	122.26	32.13	121
SBP	121.22	15.33	120
DBP	76.36	10.03	78
BMI	28.18	6.38	27

Notes: BMI: Body Mass Index; DBP: Diastolic Blood Pressure; SBP: Systolic Blood Pressure; LDL: Low-density lipoprotein; HDL: High-density lipoprotein; SD: Standard Deviation.

**Table 2:** Comparison in characteristics of subjects with and without CV events during 3 years follow-up.

Variables	Subjects with CV events			Subjects without CV events			*P* value
	(*n* = 19,445; 20.7%)			(*n* = 74,507; 79.3%)			
	Mean	SD	Median	Mean	SD	Median	
Mean followup by CV event	510.08	313.36	487	1095	—	—	
Age	44.44	9.4	45	41.39	9.66	41	<.001
Female	63%			68%			<.001
HR	76.3	11.32	76	75.7	10.89	76	<.001

Notes: CV: Cardiovascular; HR: Heart Rate; SD: Standard Deviation.

**Table 3:** Hazard ratio for Cox regression of CV events during 3-year followup (*n* = 93,952).

Variable	Hazard ratio	SE	*P* value	95% CI	
HR	1.002	0.001	.000	1.001	1.003
Age	1.024	0.001	.000	1.023	1.026
Gender					
Male	Base group				
Female	0.869	0.011	.000	0.846	0.891
Race					
White	Base group				
Black	1.381	0.041	.000	1.303	1.464
Hispanic	1.419	0.069	.000	1.289	1.561
Others	0.996	0.051	.945	0.902	1.101
Unknown	1.048	0.015	.001	1.019	1.077
Smoking status					
Current	Base group				
Former	0.970	0.025	.227	0.922	1.019
Never	0.946	0.021	.012	0.906	0.988
Unknown	0.939	0.019	.002	0.902	0.977
Payment status					
Commercial	Base group				
Medicare	0.986	0.026	.599	0.936	1.039
Medicaid	0.977	0.044	.607	0.895	1.067
Selfpay	1.019	0.055	.722	0.917	1.133
Unknown	1.037	0.014	.010	1.009	1.065
Comorbid index					
CCI	1.487	0.011	.000	1.466	1.508
CDS	1.224	0.004	.000	1.216	1.231

## References

[1] Andersson KE, Olshansky B (2007). Treating patients with overactive bladder syndrome with antimuscarinics: heart rate considerations. *BJU International*.

[2] Jouven X, Empana JP, Escolano S (2009). Relation of heart rate at rest and long-term (>20 years) death rate in initially healthy middle-aged men. *American Journal of Cardiology*.

[3] Olshansky B, Ebinger U, Brum J, Egermark M, Viegas A, Rekeda L (2008). Differential pharmacological effects of antimuscarinic drugs on heart rate: a randomized, placebo-controlled, double-blind, crossover study with tolterodine and darifenacin in healthy participants ≥50 years. *Journal of Cardiovascular Pharmacology and Therapeutics*.

[4] Kannel WB, Kannel C, Paffenbarger Jr. RS, Cupples A (1987). Heart rate and cardiovascular mortality: the Framingham study. *American Heart Journal*.

[5] De Groot V, Beckerman H, Lankhorst GJ, Bouter LM (2003). How to measure comorbidity: a critical review of available methods. *Journal of Clinical Epidemiology*.

[6] Charlson M, Szatrowski TP, Peterson J, Gold J (1994). Validation of a combined comorbidity index. *Journal of Clinical Epidemiology*.

[7] de Lissovoy G, Lazarus SS (1994). The economic cost of migraine. Present state of knowledge. *Neurology*.

[8] Charlson ME, Charlson RE, Peterson JC, Marinopoulos SS, Briggs WM, Hollenberg JP (2008). The Charlson comorbidity index is adapted to predict costs of chronic disease in primary care patients. *Journal of Clinical Epidemiology*.

[9] Chaudhry S, Jin L, Meltzer D (2005). Use of a self-report-generated Charlson comorbidity index for predicting mortality. *Medical Care*.

[10] Petersen LA, Pietz K, Woodard LD, Byrne M (2005). Comparison of the predictive validity of diagnosis-based risk adjusters for clinical outcomes. *Medical Care*.

[11] Von Korff M, Wagner EH, Saunders K (1992). A chronic disease score from automated pharmacy data. *Journal of Clinical Epidemiology*.

[12] Clark DO, Von Korff M, Saunders K, Baluch WM, Simon GE (1995). A chronic disease score with empirically derived weights. *Medical Care*.

[13] Von Korff M, Wagner EH, Saunders K (1992). A chronic disease score from automated pharmacy data. *Journal of Clinical Epidemiology*.

[14] McGregor JC, Kim PW, Perencevich EN (2005). Utility of the Chronic Disease Score and Charlson Comorbidity Index as comorbidity measures for use in epidemiologic studies of antibiotic-resistant organisms. *American Journal of Epidemiology*.

[15] Baser O, Palmer L, Stephenson J (2008). The estimation power of alternative comorbidity indices. *Value in Health*.

[16] Putnam KG, Buist DSM, Fishman P (2002). Chronic disease score as a predictor of hospitalization. *Epidemiology*.

[17] Johnson RE, Hornbrook MC, Nichols GA (1994). Replicating the chronic disease score (CDS) from automated pharmacy data. *Journal of Clinical Epidemiology*.

[18] Andersson KE, Sarawate C, Kahler KH, Stanley EL, Kulkarni AS (2010). Cardiovascular morbidity, heart rates and use of antimuscarinics in patients with overactive bladder. *BJU International*.

[19] Levine HJ (1997). Rest heart rate and life expectancy. *Journal of the American College of Cardiology*.

[20] Benetos A, Thomas F, Bean K, Albaladejo P, Guize L, Palatini P (2003). Resting heart rate in older people: a predictor of survival to age 85. *Journal of the American Geriatrics Society*.

[21] Gillum RF, Makuc DM, Feldman JJ (1991). Pulse rate, coronary heart disease, and death: the NHANES I epidemiologic follow-up study. *American Heart Journal*.

[22] Diaz A, Bourassa MG, Guertin MC, Tardif JC (2005). Long-term prognostic value of resting heart rate in patients with suspected or proven coronary artery disease. *European Heart Journal*.

[23] Palatini P, Benetos A, Julius S (2006). Impact of increased heart rate on clinical outcomes in hypertension: implications for antihypertensive drug therapy. *Drugs*.

[24] Fox K, Borer JS, Camm AJ (2007). Resting heart rate in cardiovascular disease. *Journal of the American College of Cardiology*.

[25] Palatini P, Benetos A, Julius S (2006). Impact of increased heart rate on clinical outcomes in hypertension: implications for antihypertensive drug therapy. *Drugs*.

[26] Lash TL, Mor V, Wieland D, Ferrucci L, Satariano W, Silliman RA (2007). Methodology, design, and analytic techniques to address measurement of comorbid disease. *Journals of Gerontology. Series A*.

[27] Iezzoni LI (1997). Assessing quality using administrative data. *Annals of Internal Medicine*.

